# Assessing mental health literacy in Jordan: a factor analysis and Rasch analysis study

**DOI:** 10.3389/fpubh.2024.1396255

**Published:** 2024-07-01

**Authors:** Walid Al-Qerem, Anan Jarab, Maher Khdour, Judith Eberhardt, Fawaz Alasmari, Alaa Hammad, Ruba Zumot, Sarah Khalil

**Affiliations:** ^1^Faculty of Pharmacy, Al-Zaytoonah University of Jordan, Amman, Jordan; ^2^College of Pharmacy, Al Ain University, Abu Dhabi, United Arab Emirates; ^3^Faculty of Pharmacy, Jordan University of Science and Technology, Irbid, Jordan; ^4^College of Pharmacy, Al-Quds University, Abu Dis, Palestine; ^5^School of Social Sciences, Humanities and Law, Department of Psychology, Teesside University, Middlesbrough, United Kingdom; ^6^College of Pharmacy, King Saud University, Riyadh, Saudi Arabia

**Keywords:** mental health, Jordan, literacy, Arabic, validation, conceptualization

## Abstract

**Background:**

Mental health literacy (MHL) research in Jordan is sparse and validated MHL measures are lacking. The present study validated a Jordanian version of the Mental Health Literacy Scale (MHLS) and examined Jordanian individuals’ MHL.

**Method:**

A Google Forms survey was designed, and the link was shared through various Jordanian social media platforms. Factor analysis and Rasch analysis were performed to validate the Jordanian version of the MHLS. Binary logistic regression was performed to assess variables associated with MHL.

**Results:**

The Jordanian MHLS was administered to 974 participants (74.4% females; median age 27 years). The mean MHL score of the participants was 71.1% indicating average literacy levels. The factor analysis indicated that 27 items distributed across four factors had the best model fit. The Rasch analysis confirmed item separation reliability and person reliability. The regression showed a correlation between educational attainment, income, marital status and MHL level. These findings emphasize the role of educational attainment in MHL, pointing to the necessity of integrating mental health education into formal curricula to enhance MHL across all societal levels. Stigma and limited-service availability act as barriers to mental health service and access, which compound the challenge of improving MHL. Targeted educational interventions and policy reforms may help improve MHL, thereby contributing to improving mental health outcomes in Jordan and potentially other similar settings.

## 1 Introduction

For The concept of Mental Health Literacy (MHL), first introduced in 1997, refers to the capability to identify mental health conditions and the beliefs about their therapeutic effectiveness (1). MHL involves six main components: (1) the capability to identify features of a particular mental health condition; (2) background information and beliefs about the causes of and risk factors for mental health conditions; (3) background knowledge and beliefs about self-care interventions; (4) knowledge and beliefs about the professional help available; (5) attitudes that aid in mental health condition identification and help-seeking; and (6) knowledge about how to search for related information (2).

Approximately 970 million individuals worldwide, or 1 in every 8 people, suffer from a mental illness, with anxiety and depressive disorders being the most prevalent (3). According to a systematic review of research studies from Asia and Europe, 33.7, 31.9, and 29.6% of the general population suffered from depression, anxiety, and stress, respectively, during the COVID-19 pandemic (4). About one in every seven adolescents has experienced a mental health condition, and almost 40% of the experienced mental disorders are depressive and anxiety disorders, 20.1% are conduct disorders, and 19.5% are attention deficit hyperactivity disorder (5). Many studies have been carried out to evaluate MHL in adults with results indicating a low degree of MHL in the population. Most individuals struggle to recognize mental health conditions and are uncertain how to access information about these (6).

Large community-based studies reported a high prevalence of mental illness in the Middle East, ranging from 15.6 to 35.5%, with higher rates found in countries experiencing complex emergencies like food shortages and war (7). In Jordan, an estimated 1.75 million individuals, or roughly 20% of the country’s population, suffer from mental illnesses, primarily anxiety and depression (8). According to a study conducted to quantitatively assess the burden of mental health conditions in the Eastern Mediterranean region between 1990 and 2013, there is an increasing prevalence of chronic disorders, including mental illnesses, in the region. Nearly all countries in the region experienced a higher burden of mental health disorders compared to the global average. Although this burden affected all age groups, the highest incidence was observed in the 25–49 age group (9). These statistics clearly indicate a concerning prevalence of mental disorders in the Middle East and Jordan, compared to the global estimation, highlighting the pressing need to focus on mental health-related issues in future research.

Recent studies have frequently highlighted the negative impact of insufficient health literacy, including increased incidence of chronic illnesses, elevated medical expenses, lower utilization of medical services, and earlier mortality (10). Poor MHL is considered problematic since it not only represents a behavioral health intervention barrier but also contributes to mental health stigma. This can create barriers to treatment and reduce the likelihood of individuals engaging in mental health therapy (11). According to a longitudinal, population-based study, poor MHL and negative behaviors toward mental health care were found to be associated with a reduced degree of behavioral health engagement even after controlling for sociodemographic characteristics (12). Moreover, a systematic review examining parents’ and caregivers’ MHL, found that a lack of MHL posed a barrier to accessing behavioral health therapy, and was associated with factors such as knowledge and financial barriers, disbelief in and fear of treatment services, as well as the negative reputation of mental health care (13). In the East Mediterranean region; stigmatizing attitudes toward mental illnesses were reported in a study conducted on the Lebanese population (14). In Turkey, healthcare professionals have also shown less than optimal levels of MHL, with age, marital status, education level, and occupation being the core influencing factors (15).

Sociocultural factors can have an important role in determining MHL in a specific group or population. A study conducted among Australian youth highlighted several social and cultural factors affecting MHL (16). For example, it was discovered that, in comparison to males, females are more likely to have elevated levels of MHL in relation to generalized anxiety disorders, and are noticeably more likely to recognize depression than males (17–20). In addition, a number of sociodemographic factors, such as level of education, age, and family history of mental illness, were found to be significantly correlated with MHL (21). Furthermore, because of the stigma associated with mental health clinics, the majority of patients who visit these clinics either do not use the services provided or do not follow treatment plans as required (22). In Jordan, it was found that stigma accounted for 41% of the reasons why mentally ill patients stopped attending mental healthcare clinics, postponed appointments, showed noncompliance to treatment, or showed delayed improvement (22). In Jordanian society, mental health issues are frequently stigmatized. Factors contributing to this stigma include labeling symptoms as “mental illness,” attributing these issues to biological causes, perceiving individuals with mental health conditions as dangerous, and various sociodemographic influences (22). This stigma can make people reluctant to seek assistance or openly discuss their mental health, negatively impacting their MHL.

Despite the existence of numerous studies evaluating MHL in the general population around the globe, and the availability of MHL questionnaires in various languages, there is limited numbers of studies in the Middle East, as well as a scarcity of questionnaires validated in Arabic for assessing MHL. There were two previous validations of an Arabic version of Mental Health Literacy Scale (MHLS) (23), one of them was conducted on university students (24) which casts doubt on the generalizability of the findings, and both of them were conducted in Saudi Arabia (24, 25) and therefore may not be suitable for other Arabic speaking populations including those in Levante and the East Mediterranean region, furthermore, these validations lacked important statistical analysis including Rasch analysis. Therefore, the present study aimed to validate an Arabic tool to assess MHL among the Jordanian population and evaluate MHL in the Jordanian general population. Findings of this study can be used to guide focused educational campaigns and policy development aiming at addressing knowledge gabs, and promoting early detection, intervention, and community support with regard to MHL. A more supportive and health-literate society can result from improved MHL, which can also lead to better use of mental health services and better public attitudes.

## 2 Method

A questionnaire was designed using Google Forms and the link was distributed via various Jordanian social media platforms including Jordanian webpages, and Jordanian Facebook and WhatsApp groups. The data were collected from October to December 2023. The inclusion criteria were being a resident of Jordan, aged 18 years or older, and consenting to participate in the study. Questions about the age and residency in addition to a consent section were included in the questionnaire to confirm the participants’ eligibility. The questionnaire was designed so that respondents who did not meet the stated inclusion criteria were automatically directed to the end of the form, furthermore, to avoid missing data, all the relevant questions were marked as mandatory, so that the participants were not permitted to submit the questionnaire before completing these questions. The introduction of the questionnaire presented the aim of the study and an informed consent form. Ethical approval was obtained from the Al-Zaytoonah University of Jordan ethical committee. Also, this study was conducted in line with the Declaration of Helsinki ethical principles.

### 2.1 Study instruments

After thoroughly reviewing various MHL questionnaires, the MHLS was chosen for its comprehensive coverage of different medical conditions and its use of simplified terminology (23). Upon receiving permission from the questionnaire’s author, the questionnaire was translated into Arabic (Jordan). The translation process adhered to Brislin principle (26) to assure producing an Arabic version of the questionnaire that retains the original meaning and adopts cultural relevance. Two Arabic versions of the questionnaire were produced by two independent translators, which were subsequently compared by the translators and the researchers and any discrepancy between the two versions were evaluated and modified to produce the first draft of the Arabic version of the questionnaire. Then the first draft of Arabic version was back translated by two different translators, the two back translated versions, the original questionnaire and the Arabic draft were evaluated by the researchers and translators and the final Arabic version was produced. This process ensured the accuracy and cultural appropriateness of the final Arabic version.

The MHLS consists of 35 questions assessing knowledge of mental health. It includes six attributes: the first attribute assesses the ability to identify disorders and includes eight items. The second attribute, consisting of two items, aims to evaluate knowledge regarding the causes and risk factors associated with developing mental illnesses. The third attribute, which comprises two questions, is designed to assess knowledge about self-treatment options. The fourth attribute, with three items, evaluates understanding of mental health care professionals and the services they offer. The fifth attribute, containing four items, measures the participant’s knowledge on how to seek information about mental health. The sixth and final attribute focuses on practices that promote the recognition of mental disorders and encourage help-seeking behavior, including 16 items (1). The first 15 items of the questionnaire use a 4-point Likert scale with answer options ranging from ‘very unlikely’ to ‘very likely’ or from ‘very unhelpful’ to ‘very helpful’. The remaining items employ a 5-point Likert scale, with the answer choices ‘strongly disagree’ to ‘strongly agree’ or ‘definitely unwilling’ to ‘definitely willing’. The total score is determined by summing the scores of all items. For 4-point Likert scale items, scores range from 1, representing ‘unlikely’ or ‘unhelpful’, to 4, indicating ‘very likely’ or ‘very helpful’. In the case of 5-point scale items, scores extend from 1 for ‘strongly disagree’ or ‘definitely unwilling’ to 5 for ‘strongly agree’ or ‘definitely willing’. Reverse coding was applied to negative items. Thus, the maximum score is 160 while the minimum is 35. In addition to the original questionnaire, a data collection sheet was developed to record demographical information about the participants, including their age, gender, educational qualification, income, and marital status.

### 2.2 Sample size calculation

When employing factor analysis, it is recommended to maintain a participant-to-item ratio of up to 20:1 to determine the sample size (27). Since the MHLS contains 35 items, the minimum sample size was determined to be 700.

### 2.3 Tool validation

Content validity of the MHLS was assessed by a group of experts composed of two psychologists and a clinical pharmacist. The experts confirmed that the questionnaire is comprehensive and covers different medical conditions using simplified easy to understand terminology. A pilot study was conducted to evaluate the face validity of the questionnaire with 30 Jordanian participants. The participants were randomly selected and approached in different pharmacies from different geographical areas in Jordan, the aim of the study was explained to the participants by the research assistants and a consent form was presented to them after ensuring that the participants met the study’s inclusion criteria. The participants were asked to complete the questionnaire and provide their feedback. An open discussion was conducted between the participants and the research assistants that gathered the participant’s feedback about the items’ relevance, ease of response, and items clarity. The feedback was discussed by the researchers, experts and translators and minor changes were made based on the feedback by using synonyms of 6 words. The pilot study data was not included in the statistical analysis for the present study. Moreover, confirmatory factor analysis (CFA) and principal component analysis (PCA) were performed to assess the construct validity of the questionnaire. Factor analysis is a valid and widely used method the generate the most accurate construct from the participants’ responses to MHLS, it confirms the relation between the observed variables and the latent ones and simplifies the interpretation of the data by grouping the observed variables into fewer latent ones. The internal consistency of each generated factor was assessed using Cronbach’s alpha. Rasch analysis was conducted to determine the tool’s ability to differentiate between different participants’ health literacy levels and to assess the difficulty levels of the questionnaire’s items. Generally, Rasch analysis is applied to evaluate the quality of instruments with intendent latent variables. Wright maps, item fit, person fit and ability, and instrument analyses are inspected to evaluate the tool quality (28). To confirm the validity of the instrument in Rasch analysis, observed data are expected to fit the probabilistic relationship within and between person estimates and item estimates as specified in the Rasch measurement model (29).

### 2.4 Statistical analysis

Statistical analyses were conducted using R software with the Test Analysis Modules (TAM) package version 4.1–4, and the Statistical Package for the Social Sciences (SPSS) version 26. Bartlett’s Test and Kaiser-Meyer-Olkin (KMO) analysis were used to determine the suitability of the data for performing PCA. The scree plot was examined, and parallel analysis was performed to determine the appropriate number of factors to be extracted. The direct-oblimin rotation method was used to generate a pattern matrix. Communalities were assessed, and any item with a value lower than 0.3 was not included in the analysis. Factors loadings were also assessed and any item with loadings lower than 0.4 or with multiple factor loadings greater than 0.4 was excluded from the analysis. A multi-factorial Rasch analysis for polytomous responses was performed. Two reliability measures, Person Separation and Item Separation, were calculated to confirm the suitability of the model. Furthermore, infit/outfit statistics were generated. Infit and outfit mean square (MSQ) values between 0.6 and 1.4 were considered acceptable (30). Thresholds were computed to evaluate each item, and a Wright map was produced. Two Differential Item Functioning (DIF) analyses between genders were conducted, one for the Recognition factor and the second for the remaining factors, due to the differences in the response scale between the Recognition factor and the remaining ones it could not be included in the same DIF analysis. A DIF value of <0.43 logits is considered acceptable (31).

The participants were categorized into low and high MHL groups. The high MHL group included participants who scored above the mean of the MHLS, while the rest were included in the low MHL group. Subsequently, the MHL groups were included as an outcome variable in a binary regression model which also included age, gender, education and income levels, and marital status as predictor variables. Statistical significance was established at *p* < 0.05.

## 3 Results

The present study enrolled 982 participants, with 74.5% being female. Demographic details of the participants are presented in Table 1. The median age of the participants was 27, with an age range from 21 to 35. Regarding education levels, 19% of the participants had completed only high school, 10.8% held diplomas, and 64.8% had obtained bachelor’s degrees. Additionally, 5.6% possessed postgraduate degrees. In terms of marital status, 36.6% were married. Furthermore, 59.7% reported a monthly income of less than 500 Jordanian Dinars.

**Table 1:** Participants’ socio-demographic characteristics.

		Median (Percentile 25–75)	Count (%)
Age		27 (21–35)	
Gender	Female		732 (74.5%)
	Male		250 (25.5%)
Educational level	High school or less		187 (19%)
	Diploma		106 (10.8%)
	Bachelor’s degree		634 (64.8%)
	Higher education		55 (5.6%)
Marital status	Other		623 (63.4%)
	Married		359 (36.6%)
Income	Less than 500		586 (59.7%)
	Between 500–100		263 (26.8%)
	More than 1,000		133 (13.5%)

**Figure 1:**
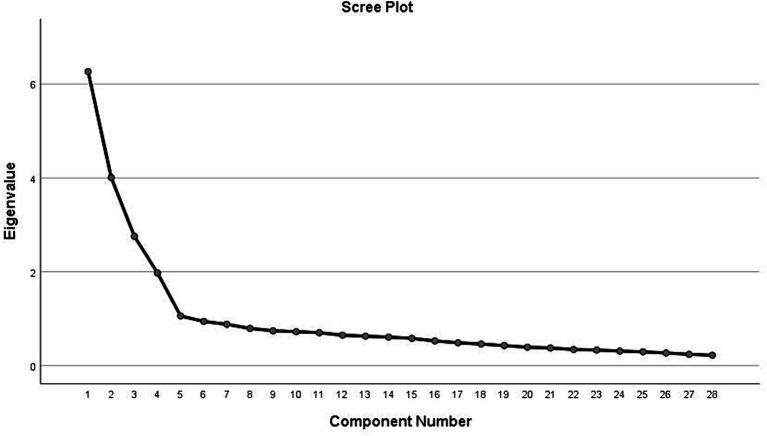
Scree plot of factor analysis.

KMO and Bartlett’s test of sphericity indicated that the data were suitable for factor analysis (KMO = 0.882, *p* < 0.001). Scree plots (Figure 1) and parallel analysis suggested a four-factor solution. Items with low communalities were deleted, including “1-If someone became extremely nervous or anxious in one or more situations with other people (e.g., a party) or performance situations (e.g., presenting at a meeting) in which they were afraid of being evaluated by others and that they would act in a way that was humiliating or feel embarrassed, then to what extent do you think it is likely they have Social Phobia?,” “3-If someone experienced a low mood for two or more weeks, had a loss of pleasure or interest in their normal activities and experienced changes in their appetite and sleep then to what extent do you think it is likely they have Major Depressive Disorder?,” “9-To what extent do you think it is likely that in general in Jordan, women are MORE likely to experience a mental illness of any kind compared to men?,” “12-To what extent do you think it would be helpful for someone to avoid all activities or situations that made them feel anxious if they were having difficulties managing their emotions?,” 15- “Mental health professionals are bound by confidentiality; however there are certain conditions under which this does not apply. To what extent do you think it is likely that the following is a condition that would allow a mental health professional to break confidentiality: if your problem is not life-threatening and they want to assist others to better support you?,” “10-To what extent do you think it is likely that in general, in Jordan, men are MORE likely to experience an anxiety disorder compared to women?,” 14- “Mental health professionals are bound by confidentiality; however there are certain conditions under which this does not apply. To what extent do you think it is likely that the following is a condition that would allow a mental health professional to break confidentiality: If you are at immediate risk of harm to yourself or others?” and “20-People with a mental illness could snap out of it if they wanted.”

Table 2 depicts factor loadings, communalities, and Cronbach’s alpha for the questionnaire items. The produced factors included “Recognition,” “Information seeking,” “General attitude” and “Attitudes Toward People.” For the General Attitudes Factor, loadings values ranged from 0.827 to 0.714 while the communalities ranged from 0.704 to 0.542 with a Cronbach’s alpha value of 0.913. For the Attitudes Toward People Factor, loadings ranged from 0.817 to 0.646 while communalities ranged from 0.682 to 0.540 with a Cronbach’s alpha factor of 0.866, the Recognition Factor loadings ranged from 0.648 to 0.576 while communalities ranged from 0.443 to 0.338 with a Cronbach’s alpha value of 0.761, and the Information-Seeking Factor loadings ranged from 0.782 to 0.569 whereas communalities varied from 0.635 to 0.395 with a Cronbach’s alpha value of 0.750.

**Table 2:** Loadings, communalities, and Cronbach’s alpha values.

	Mean (SD)	loadings	Communalities	Factor’s Cronbach’s alpha
**Recognition**				
2-If someone experienced excessive worry about a number of events or activities where this level of concern was not warranted, had difficulty controlling this worry and had physical symptoms such as having tense muscles and feeling fatigued then to what ext	3 (1)	0.576	0.362	0.761
4-To what extent do you think it is likely that Personality Disorders are a category of mental illness	4 (1)	0.631	0.400	
5-To what extent do you think it is likely that Dysthymia is a disorder	4 (1)	0.638	0.444	
6-To what extent do you think it would be helpful for someone to improve their quality of sleep if they were having difficulties managing their emotions (e.g., becoming very anxious or depressed)	3 (1)	0.592	0.340	
7-To what extent do you think it is likely that the diagnosis of Bipolar Disorder includes experiencing periods of elevated (i.e., high) and periods of depressed (i.e., low) mood	3 (1)	0.648	0.409	
8-To what extent do you think it is likely that the diagnosis of Drug Dependence includes physical and psychological tolerance of the drug (i.e., require more of the drug to get the same effect)	3 (1)	0.576	0.360	
11-To what extent do you think it is likely that the diagnosis of Agoraphobia includes anxiety about situations where escape may be difficult or embarrassing	3 (1)	0.591	0.340	
13-To what extent do you think it is likely that Cognitive Behavior Therapy (CBT) is a therapy based on challenging negative thoughts and increasing helpful behaviors	3 (1)	0.586	0.350	
**Information seeking**				
16-I am confident that I know where to seek information about in mental illness	4 (1)	0.741	0.670	0.750
17-I am confident using the computer or telephone to seek information about mental illness	4 (1)	0.711	0.506	
18-I am confident attending face to face appointments to seek information about mental illness (e.g., seeing the GP)	4 (1)	0.667	0.548	
19-I am confident I have access to resources (e.g., GP, internet, friends) that I can use to seek information about mental illness	4 (1)	0.784	0.628	
**General attitude**				
21-If I had a mental illness, I would not seek help from a mental health professional	3 (1)	0.783	0.612	0.913
22-A mental illness is not a real medical illness	3 (1)	0.719	0.603	
23-If I had a mental illness I would not tell anyone	3 (1)	0.749	0.541	
24-It is best to avoid people with a mental illness so that you do not develop this problem	3 (1)	0.814	0.686	
25-People with a mental illness are dangerous	3 (1)	0.742	0.566	
26-Seeing a mental health professional means you are not strong enough to manage your own difficulties	3 (1)	0.831	0.706	
27-A mental illness is a sign of personal weakness	3 (1)	0.782	0.675	
28-I believe treatment for a mental illness, provided by a mental health professional, would not be effective	3 (1)	0.792	0.679	
**Attitudes toward people**				
29-How willing would you be to have someone with a mental illness marry into your family?	3 (1)	0.655	0.542	0.866
30-How willing would you be to spend an evening socializing with someone with a mental illness?	4 (1)	0.783	0.604	
31-How willing would you be to make friends with someone with a mental illness?	4 (1)	0.791	0.683	
32-How willing would you be to have someone with a mental illness start working closely with you on a job?	3 (1)	0.824	0.653	
33-How willing would you be to employ someone if you knew they had a mental illness?	3 (1)	0.685	0.665	
34-How willing would you be to vote for a politician if you knew they had suffered a mental illness?	3 (1)	0.640	0.585	
35-How willing would you be to move next door to someone with a mental illness?	3 (1)	0.707	0.545	

In terms of means and standard deviations, the mean of all items on the General Attitudes Factor were 3 (SD = 1). In the Attitudes Toward People Factor, the highest mean score was 4 (SD = 1), observed for two items: “31-How willing would you be to make friends with someone with a mental illness?” and “30-How willing would you be to spend an evening socializing with someone with a mental illness?” The mean scores for the remaining items in this factor were 3 (SD = 1). In the Recognition Factor, the highest mean score was 4 (SD = 1), noted for two items: “5-To what extent do you think it is likely that Dysthymia is a disorder?” And “4-to what extent do you think it is likely that Personality Disorders are a category of mental illness?” The lowest mean score within this factor was 3 (SD = 1), which was recorded for the six remaining items. In the Information-Seeking Factor, all items had a uniform mean score of 4 (SD = 1). The mean for the Recognition score was 26.60 (SD = 3.80), and the interquartile range was 4.25. For the Information seeking score the mean was 15.75 (SD = 3.02), and the interquartile range was 4.00, while in the General attitude scale the mean was 25.50 (SD = 7.57), and the interquartile range was 12. Lastly in Attitude toward people score the mean was 22.61 (SD = 5.78), and the interquartile range was 8. The mean MHLS score for all participants was 90.4 (SD = 10.5) out of a maximum possible score of 127.

**Figure 2:**
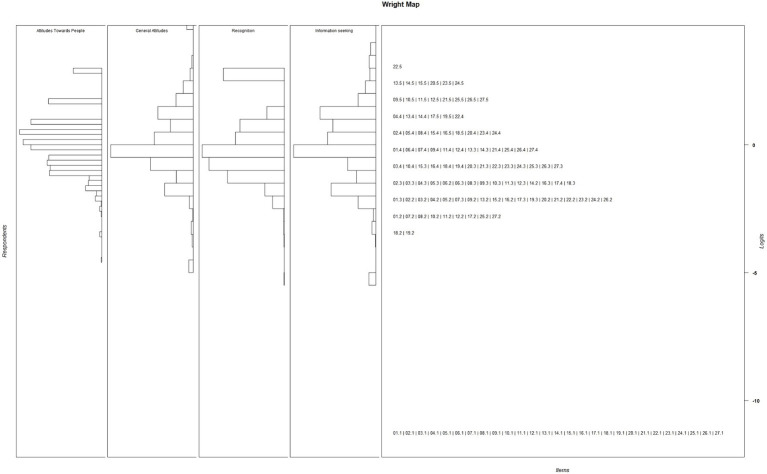
Wright map produced by Rasch analysis.

The Rasch model analysis indicated that the item separation reliability and person reliability for the General Attitudes, Attitudes Toward People, Recognition, and Information-Seeking Factors were 0.727 and 0.644, 0.868 and 0.853, 0.738 and 0.638, and 0.905 and 0.887, respectively. Infit and outfit MSQ values are presented in Table 3. None of the items violated the acceptable range of Infit and outfit MSQ. The thresholds displayed in Table 3 indicate that all items had ordered response categories. The Wright map, shown in Figure 2, confirms that the patients’ responses were distributed across all difficulty levels within the four factors. The distribution of item thresholds across various difficulty levels revealed differing levels of challenge for participants. The lowest difficulty score was −11.34 in the first category, observed in 15 items, whereas the highest difficulty score was associated with the item “25-People with a mental illness are dangerous” in the fifth category, which had a value of 3.23.

**Table 3:** Outfits, infits, and thresholds of the MHLS items.

Items	Outfit	Infit	Thresholds				
			1	2	3	4	5
2-If someone experienced excessive worry about a number of events or activities where this level of concern was not warranted, had difficulty controlling this worry and had physical symptoms such as having tense muscles and feeling fatigued then to what extent do you think it is likely they have Generalized Anxiety Disorder	0.94	0.95	−11.29	−2.62	−1.94	−0.05	NA
4-To what extent do you think it is likely that Personality Disorders are a category of mental illness	0.97	0.97	−11.33	−2.29	−1.51	0.31	NA
5-To what extent do you think it is likely that Dysthymia is a disorder	0.85	0.91	−11.33	−2.22	−1.60	−0.62	NA
6-To what extent do you think it is likely that the diagnosis of Agoraphobia includes anxiety about situations where escape may be difficult or embarrassing	0.97	0.98	−11.33	−2.44	−1.53	0.80	NA
7-To what extent do you think it is likely that the diagnosis of Bipolar Disorder includes experiencing periods of elevated (i.e., high) and periods of depressed (i.e., low) mood	0.96	0.96	−11.33	−2.32	−1.54	0.36	NA
8-To what extent do you think it is likely that the diagnosis of Drug Dependence includes physical and psychological tolerance of the drug (i.e., require more of the drug to get the same effect)	1.00	1.02	−11.34	−1.72	−1.42	−0.37	NA
11-To what extent do you think it would be helpful for someone to improve their quality of sleep if they were having difficulties managing their emotions (e.g., becoming very anxious or depressed)	1.02	0.99	−11.28	−2.59	−1.87	−0.25	NA
13-To what extent do you think it is likely that Cognitive Behavior Therapy (CBT) is a therapy based on challenging negative thoughts and increasing helpful behaviors	0.97	0.97	−11.33	−2.51	−1.80	0.18	NA
29-How willing would you be to move next door to someone with a mental illness?	1.11	1.10	−11.34	−2.31	−1.19	−0.39	1.94
30-How willing would you be to spend an evening socializing with someone with a mental illness?	0.97	0.98	−11.30	−3.01	−1.52	−0.73	1.62
31-How willing would you be to make friends with someone with a mental illness?	0.90	0.91	−11.34	−2.56	−1.35	−0.41	1.68
32-How willing would you be to have someone with a mental illness start working closely with you on a job?	0.89	0.90	−11.34	−2.90	−1.30	−0.30	1.96
33-How willing would you be to have someone with a mental illness marry into your family?	0.91	0.91	−11.34	−1.94	−0.29	0.82	2.49
34-How willing would you be to vote for a politician if you knew they had suffered a mental illness?	1.04	1.04	−11.34	−1.66	−0.19	0.83	2.41
35-How willing would you be to employ someone if you knew they had a mental illness?	1.02	1.03	−11.34	−2.35	−0.58	0.55	2.13
16-I am confident that I know where to seek information about mental illness	0.83	0.87	−11.33	−2.15	−1.65	−1.05	0.54
17-I am confident using the computer or telephone to seek information about mental illness	1.06	1.11	−11.28	−3.07	−1.96	−1.20	0.84
18-I am confident attending face to face appointments to seek information about mental illness (e.g., seeing the GP)	0.98	1.00	−11.28	−3.26	−1.81	−1.08	0.71
19-I am confident I have access to resources (e.g., GP, internet, friends) that I can use to seek information about mental illness	0.90	0.91	−11.28	−3.60	−1.87	−0.99	0.84
21-A mental illness is a sign of personal weakness	1.00	1.01	−11.34	−2.44	−0.60	0.14	2.13
22-A mental illness is not a real medical illness	1.04	1.06	−11.34	−2.18	−0.61	0.07	1.81
23-People with a mental illness are dangerous	1.21	1.20	−11.34	−2.18	−0.53	0.85	3.23
24-It is best to avoid people with a mental illness so that you do not develop this problem	0.89	0.89	−11.34	−2.37	−0.76	0.15	2.10
25-If I had a mental illness I would not tell anyone	1.17	1.13	−11.34	−2.38	−0.85	0.47	2.67
26-Seeing a mental health professional means you are not strong enough to manage your own difficulties	0.81	0.84	−11.34	−2.66	−0.89	−0.14	1.60
27-If I had a mental illness, I would not seek help from a mental health professional	0.91	0.92	−11.34	−2.39	−0.84	−0.05	1.83
27-I believe treatment for a mental illness, provided by a mental health professional, would not be effective	0.90	0.88	−11.34	−2.49	−0.93	−0.03	1.89

It is noticed that the two genders’ locations are close. The difference between the two genders on the logit scale for the Recognition factor and for the remaining factor were found to be 0.089 and 1.1 logits respectively, which are less than the cut-off point of ≥0.43 logits, indicating that there is no significant difference between the two genders.

A binary regression model was applied to determine the association between various sociodemographic variables and MHLS scores. Participants with a high school degree or lower had significantly lower odds of having high MHLS scores compared to those with a bachelor’s degree and higher (OR = 0.366, 95% Cl (0.238–0.564), *p* < 0.001). Unmarried participants had significantly higher odds of having high MHLS scores compared to married participants [OR = 1.630, 95% Cl (1.121–2.369), *p* < 0.010]. Also, participants who reported earning less than 500 JD or 500–1,000 JD a month had significantly lower odds of being in the high MHL group compared to participants who reported earning more than 1,000 JD a month [OR = 0.241, 95% Cl (0.149–0.391), *p* < 0.001]; [OR = 0.385, 95% Cl (0.235–0.631), *p* < 0.001], respectively.

## 4 Discussion and conclusion

The current study aimed to assess mental health literacy (MHL) in Jordan, focusing on individuals’ knowledge, attitudes, and behaviors toward mental health, and examining the influence of sociodemographic variables on these factors. The findings evidenced the Jordanian version of the MHLS’s ability to differentiate across a range of mental health literacy competencies among participants. The data were suitable for factor analysis, which confirms the robustness of the translated MHLS in capturing diverse aspects of mental health literacy in the Jordanian participants. The refinement of the scale through item deletion improved its precision. The identified factors, along with their reliability scores and item distribution, demonstrate the questionnaire’s effectiveness in discerning various levels of mental health literacy across participants.

The best model for the present data was a 27-item model distributed across four factors; a similar number of factors was suggested by O’Connor and Casey’s original research (23). As they stated that the most viable structure was a 4-factor structure, however, they had low communalities and mean factor loadings. Therefore, instead of deleting these items, as we did in the present study, the authors decided to retain a univariate structure with all 35 items. However, in the present study these items were deleted to produce a more suitable, valid, and precise model; this was also done for the Iranian adoption of the questionnaire which deleted six items (1), the Slovenian version which omitted eight items (32), the Persian version which omitted five items (33), and six items were deleted in the Chinese version (34). The four-factor solution was also suggested for the Slovenian and Chinese versions (32, 34).

Moreover, although eight items were deleted, the questionnaire retained items for all the attributes of the original questionnaire: 1-Knowledge of information-seeking for mental health disorders, 2- mental disorders recognition ability, 3-knowledge of self-treatment, 4-attitudes that promote recognition or appropriate help-seeking behavior, and 5-knowledge of professional help available, except for knowledge of risk factors and causes, which had only two items asking about the association between sex and mental disorders, and thus cannot produce a reasonable assessment of knowledge about mental disorders risk factors. Moreover, the incidence of anxiety between genders may be different in different countries (17). Other items that were deleted in the present study included item 20, which was also omitted from other adaptations including the Chinese, Iranian, Persian and Slovenian versions (1, 32–34), which evaluates self-cure for mental health problems, but seems to be too absolute because mental illness could be self-cured in some cases, such as self-practiced mindfulness therapy (35). Item 12 was also deleted from the Slovenian and Persian versions and item 14 from the Slovenian version. These variations between modifications can be due to cultural and linguistic differences and/or due to the statistical analyses applied (32).

The factors produced in the present study were “Attitudes Toward People,” “General Attitudes,” “Recognition” and “Information-Seeking,” which are identical to the factors produced in the Slovenian study with similar item distributions between these factors (32).

The “General Attitudes” factor captures prevailing societal perceptions that might either hinder or promote the pursuit of mental health care. In the Jordanian context, where community opinion often profoundly influences individual behavior, these general attitudes could lead to the stigmatization of those experiencing mental health issues, thus discouraging them from seeking help. A previous study on the paradox of self-stigma in mental illness (36) revealed how stigmatizing attitudes, both internally and externally perceived, can deter individuals across various cultures from utilizing mental health services, highlighting a widespread barrier that needs addressing through targeted public awareness programs.

The “Attitudes Toward People” factor indicates the level of societal willingness to interact and empathize with individuals suffering from mental health issues. This factor is particularly central in Middle Eastern societies, where strong family ties and community bonds play a significant role (37). A study conducted in Jordan surveying adult perceptions of mental health treatment for Arab adolescents with depression, revealed no increased stigma against those seeking treatment. Instead, it showed a societal endorsement for mental health interventions, perceived equally beneficial for both genders (38). Such findings suggest shifting public attitudes toward greater acceptance and support for mental health issues, highlighting a decrease in stigma associated with receiving treatment.

The “Recognition” factor encompasses the ability to correctly identify mental health conditions, which is essential for early detection and intervention. It has been argued that recognition is the first crucial step toward effective mental health care (6). In Jordan, enhancing recognition capabilities through both formal education and public health initiatives could significantly improve early intervention strategies, thus addressing one of the primary barriers to effective mental health care identified in this region.

The “Information-Seeking” factor highlights the importance of accessing reliable and culturally appropriate information for managing one’s mental health. Participants in our study who actively sought information were more likely to engage in preventive measures and seek early treatment. This finding is supported by research noting that improved access to mental health information fosters proactive health behaviors and enhances overall mental health literacy (39).

The present study’s findings align with several international studies while also highlighting unique cultural nuances. For instance, similar to findings in an Australian context, our results indicate a general recognition of symptoms associated with common mental disorders. However, unlike the Australian adaptation, the Jordanian population demonstrated a more significant influence of cultural stigma and familial expectations on mental health perceptions and behaviors. This divergence underlines the significant impact of cultural norms prevalent in Middle Eastern societies, where mental health issues are often stigmatized and mental health care is heavily influenced by family opinions (38).

Further, while studies like (39) in Canada have shown high levels of public willingness to engage with mental health education, our findings suggest a more reserved acceptance in Jordan, possibly due to societal stigma and traditional beliefs about mental health. This aligns with observations in Arab societies, where community and family judgments significantly affect individual health-seeking behavior (40).

The present study reveals a nuanced understanding of mental health literacy compared to Western contexts (2), outlines mental health literacy in Western societies, highlighting common public misconceptions and knowledge gaps about mental health disorders. Comparing this with the current findings, both universal challenges in mental health literacy, such as widespread stigma and lack of basic knowledge, and distinct cultural differences are evident. For example, while Western contexts may exhibit higher levels of literacy regarding the symptoms and management of mental disorders, in Jordan, traditional beliefs and the role of family may significantly influence attitudes and practices around mental health. These cultural and contextual differences suggest specific implications for mental health policy and practice in Jordan. Mental health initiatives in Jordan may need to focus more on community-based interventions that respect and incorporate familial roles and address the specific stigma associated with mental health care in this region. Furthermore, our study supports the need for culturally tailored mental health education programs that consider the unique societal structures and beliefs in Jordan, similar to those suggested by Patel et al. (41) for culturally sensitive approaches in diverse settings.

The study showed various demographic factors influencing individuals’ understanding, attitudes, and behaviors in relation to mental health, specifically income, marital status, and education. Younger individuals tend to exhibit higher MHL, likely attributable to their more frequent use of digital platforms where mental health information is readily available. This generational difference in MHL suggests potential for targeted public health initiatives that leverage digital media to reach younger populations, aligning with findings that suggest a shift in mental health awareness across different age groups (39).

Furthermore, gender differences were evident in the present findings, with females generally displaying a better understanding and more positive attitudes toward mental health. This disparity may reflect the broader societal norms and gender roles that influence health-seeking behaviors, similar to patterns observed in other Middle Eastern contexts (38). Addressing these gender-specific differences is important for developing mental health programs that are sensitive to the distinct needs of both men and women.

Particularly remarkable is the impact of educational attainment on MHL. Participants with higher education levels demonstrated a more nuanced understanding of mental health issues and more positive attitudes toward mental health care. This correlation shows the importance of education as a tool in enhancing MHL, advocating for the integration of mental health education into formal curricula at all levels. Higher educational attainment is linked to higher income (42, 43) which may explain the association of income with MHL found in this study; individuals from lower income brackets exhibited lower literacy, pointing to economic barriers in accessing mental health information and services. This underscores the importance of making mental health resources accessible across all socioeconomic groups, possibly through community-based initiatives that are both affordable and widespread.

The present study identified several critical barriers to accessing mental health services in Jordan. Stigma, lack of awareness, and limited accessibility to mental health services pose significant challenges to accessing mental health services, necessitating a multifaceted approach to address these issues. A systematic review focusing on mental health literacy in the Gulf Cooperation Council countries, including Saudi Arabia, UAE, Qatar, Oman, and Kuwait, revealed limited mental health literacy among participants, even among healthcare professionals, and highlighted the presence of high levels of stigma and negative attitudes toward mental health in the public. It emphasized the need for well-designed large-scale studies and campaigns to promote early identification and treatment of mental illness to improve mental health literacy in the region (44). This review underlines the pervasive nature of stigma and negative attitudes toward mental health across the region, advocating for robust, large-scale educational campaigns aimed at enhancing early identification and treatment of mental illnesses. Furthermore, barriers to accessing mental health services in Jordan and the Eastern Mediterranean region more broadly have been reported in previous literature (9). These studies collectively suggest a regional pattern of challenges.

Stigma associated with mental health issues can deter individuals from seeking help and can be perpetuated by cultural norms, lack of awareness, and societal misconceptions. This stigma is often reinforced by media portrayals and a general lack of public discourse about mental health, which sustains a cycle of silence and misunderstanding around such issues. Stigma is manifested in various forms, from societal attitudes to self-stigma, hindering individuals from acknowledging mental health issues and seeking help. A recent systematic review of 144 studies has demonstrated the detrimental impact of mental health-related stigma on help-seeking behaviors, emphasizing the complexity of stigma as a barrier to accessing mental health care. It is suggested that addressing stigma requires a nuanced understanding of its various forms and their effects on help-seeking intentions and behaviors (45). However, although interventions aimed at enhancing mental health literacy successfully increase knowledge related to mental health, they have limited impact on diminishing stigma or fostering improvements in seeking help for mental health issues (46). Efforts to combat stigma must therefore be broad-based, involving not just education but also community engagement, to foster a more supportive and understanding societal attitude toward mental health in Jordan.

Community engagement is a low-cost, low-risk strategy that can effectively reduce stigma and social exclusion associated with mental health issues. By fostering social capital and interdependent relationships within communities, such efforts can lead to greater social cohesion and a more inclusive environment for individuals facing mental health challenges. This approach aligns with the recovery ethos in mental health care, aiming to create a supportive environment that promotes mutual support and trust (47). By involving community leaders and influencers in mental health campaigns, the messages can reach a wider audience and may be more effectively received. These leaders can help change public perceptions by openly discussing mental health, thus normalizing these conversations within the community. Additionally, involving individuals who have experienced mental health issues in these discussions can provide real-life contexts and testimonials, which can be powerful in changing attitudes.

Public awareness campaigns, supported by both traditional and social media, are another vital strategy (48). These campaigns can promote success stories of mental health treatment, highlight the availability of services, and emphasize that mental health is an integral part of overall well-being. The use of media can also help to dispel myths and build a more supportive environment for individuals experiencing mental health challenges.

Lastly, improving access to mental health services is crucial (49). This includes not only increasing the availability of services but also making them more accessible financially and geographically. Policies aimed at integrating mental health services into primary healthcare settings can reduce the stigma of seeking help and make these services more accessible to the general population. Accessibility and availability of mental health services play a crucial role in MHL and the broader mental health landscape in Jordan. The geographical distribution of services, affordability, and the quality of care available are all factors that can either facilitate or impede access to mental health care. Policies aimed at expanding the mental health care infrastructure, increasing the workforce, and ensuring the affordability of services are essential to improve access. For example, a study exploring barriers to mental health service utilization among Syrian refugees in Jordan, identified a lack of awareness of mental health issues, limited availability and accessibility of services, and stigma as major obstacles (50). A similar study highlighted financial constraints, clinician shortages, and stigma, but also noted the positive aspects of Jordan’s response, such as open-door policies and community approaches to care (51). Although these studies did not focus on the general population, these findings nevertheless emphasize the need for policymakers and healthcare providers to improve service accessibility and raise awareness to address mental health needs effectively.

The findings of the present study also draw attention to the need for targeted interventions to improve MHL among specific demographic groups. There is evidence to suggest that tailored interventions to enhance mental health literacy among specific demographic groups are effective at improving individuals’ understanding of mental health issues and encouraging more effective help-seeking behaviors, thereby enhancing the overall impact of MHL initiatives (52). Thus, such tailored programs and awareness campaigns can address the unique needs and barriers faced by different segments of the population.

### 4.1 Strengths, limitations, and future directions

This is the first study to conduct a thorough Rasch analysis on the MHLS and produce infits, outfits and a Wright map, and to confirm the item and person separation reliability of the tool. The study also included a large sample size (*n* = 974) from different sociodemographic backgrounds, which increases the validity of the study results.

This study, while providing valuable insights into mental health literacy in Jordan, is not without limitations. First, the cross-sectional design restricts the ability to draw causal inferences from the data. While valuable for identifying associations at a single point in time, this design does not allow for conclusions about the directions of these relationships. Longitudinal studies would be valuable for tracking changes in mental health literacy over time and assessing the impact of interventions.

Second, while this study covers a broad range of sociodemographic variables, the specific cultural and social nuances within Jordan that may influence mental health literacy were not extensively explored. The depth of understanding regarding how traditional beliefs, social norms, and practices impact mental health literacy is therefore limited. Future research could delve deeper into cultural factors, examining how traditional beliefs and practices impact mental health literacy. This should incorporate qualitative methods that can delve deeper into these cultural factors, potentially revealing insights that quantitative data alone cannot provide.

Additionally, the study’s focus on Jordan may limit the generalizability of the findings to other contexts. The findings may not be readily generalizable to other contexts, particularly to regions with different cultural, economic, or healthcare landscapes. Comparative studies involving multiple regions within the Middle East, or even broader international contexts, could explore the variability in mental health literacy across diverse cultural settings, enhancing external validity.

Future research should also explore the development and evaluation of targeted interventions designed to improve MHL among specific demographic groups identified in this study as having lower literacy levels. These interventions could be tailored to address the unique needs and barriers faced by these groups, enhancing their effectiveness. Moreover, the role of digital platforms in improving mental health literacy presents an area which would be useful to explore, especially in the context of the COVID-19 pandemic’s impact on mental health services accessibility.

The present findings point to the necessity of implementing culturally tailored educational programs that increase awareness of mental health issues and promote positive attitudes toward seeking help. Given the pervasive influence of stigma and the significant role of cultural and familial norms in shaping attitudes toward mental health, public awareness campaigns should be designed to address these specific barriers.

Educational initiatives could include the development of school-based programs that start from an early age to foster a foundational understanding of mental health. These could be integrated into the national curriculum to ensure they reach a broad segment of the population. Moreover, professional development for educators and healthcare providers on mental health issues could enhance the community’s capacity to support individuals facing mental health challenges.

Additionally, using media platforms to promote mental health awareness and destigmatization is vital. Campaigns could focus on disseminating success stories of individuals who have sought mental health treatment, which can serve as powerful testimonials to reduce stigma. These stories can also highlight the effectiveness of treatment, thereby challenging prevalent myths and misconceptions about mental health disorders.

The role of community leaders and religious figures in shaping public opinion cannot be overstated in the Jordanian context. Involving these influencers in mental health advocacy efforts could facilitate a change in public perceptions. Workshops and seminars to equip these leaders with basic mental health literacy could enable them to support their communities effectively.

Furthermore, improving access to mental health services involves not only increasing the availability of such services but also enhancing their affordability and accessibility. Strategies may include the integration of mental health services into primary healthcare settings, reducing stigma and making services more approachable for the general population. Policy reforms aimed at supporting mental health initiatives financially and logistically are essential to ensure that these services are sustainable.

Lastly, the development of digital mental health resources, such as mobile health applications and online therapy platforms, can play a significant role in improving access. These tools can provide valuable support, especially in areas where traditional mental health services are limited. Ensuring that these digital solutions are culturally sensitive and available in the local language will increase their effectiveness and reach.

## 5 Conclusion

This study sheds light on the varying levels of MHL in Jordan, emphasizing the influence of sociodemographic factors. The findings suggest a pressing need for targeted educational interventions to address gaps in MHL, particularly among specific demographic groups. Enhancing MHL would help foster a more supportive environment for mental health care in Jordan and potentially in similar contexts across the Middle East. Future research should continue to explore strategies for improving MHL and reducing barriers to mental health care access. While the present study contributes to the body of knowledge on mental health literacy in the Middle East, it also opens several avenues for future research to build upon these findings, aiming for a more in-depth understanding and enhancement of mental health literacy across different populations.

## Data availability statement

The datasets presented in this study can be found in online repositories. The names of the repository/repositories and accession number(s) can be found at: https://doi.org/10.5281/zenodo.10781205 Zenodo.

## Ethics statement

The studies involving humans were approved by the Al-Zaytoonah University of Jordan ethical committee. This study was conducted in line with the Declaration of Helsinki ethical principles and local legislation and institutional requirements. The participants provided their written informed consent to participate in this study.

## Author contributions

WA-Q: Conceptualization, Formal analysis, Methodology, Supervision, Validation, Writing – original draft, Writing – review & editing. AJ: Conceptualization, Investigation, Project administration, Writing – original draft, Writing – review & editing. MK: Conceptualization, Methodology, Resources, Visualization, Writing – original draft, Writing – review & editing. JE: Conceptualization, Investigation, Software, Writing – original draft, Writing – review & editing. FA: Data curation, Methodology, Project administration, Validation, Writing – original draft, Writing – review & editing. AH: Project administration, Validation, Writing – original draft, Writing – review & editing. RZ: Investigation, Resources, Writing – original draft, Writing – review & editing. SK: Resources, Visualization, Writing – original draft, Writing – review & editing.

## References

[1] Nejatian M Tehrani H Momeniyan V Jafari A. A modified version of the mental health literacy scale (MHLS) in Iranian people. BMC Psychiatry. (2021) 21:53. doi: 10.1186/s12888-021-03050-3, PMID: 33485306 PMC7824912

[2] Jorm AF. Mental health literacy. Br J Psychiatry. (2000) 177:396–401. doi: 10.1192/bjp.177.5.396 11059991

[3] WHO. (2022). *Mental disorders*. Available at: https://www.who.int/news-room/fact-sheets/detail/mental-disorders/?gad_source=1&gclid=CjwKCAjwyJqzBhBaEiwAWDRJVH2_FAfntND6HRFmvIXMN1J0CYmeIUlnRrBU4MEG24klXrBlB2O5jxoCGtkQAvD_BwE (Accessed June 10, 2024).

[4] Salari N Hosseinian-Far A Jalali R Vaisi-Raygani A Rasoulpoor S Mohammadi M. Prevalence of stress, anxiety, depression among the general population during the COVID-19 pandemic: a systematic review and meta-analysis. Glob Health. (2020) 16:57. doi: 10.1186/s12992-020-00589-w, PMID: 32631403 PMC7338126

[5] UNICEF. *Adolescent health dashboards*. (n.d.). Available at: https://data.unicef.org/resources/adolescent-health-dashboardscountry-profiles/ (Accessed January 24, 2024).

[6] Jorm AF Barney LJ Christensen H Highet NJ Kelly CM Kitchener BA. Research on mental health literacy: what we know and what we still need to know. Aust N Z J Psychiatry. (2006) 40:3–5. doi: 10.1080/j.1440-1614.2006.01734.x, PMID: 16403031

[7] Ibrahim NK. Epidemiology of mental health problems in the Middle East In: Laher I, editor. Handbook of healthcare in the Arab world. Cham: Springer International Publishing (2021). 133–49.

[8] Tayseer R. (2023). *20% of Jordanians suffer from depression, anxiety–National Centre for Mental Health Jordan Times*. Available at: https://jordantimes.com/news/local/20-jordanians-suffer-depression-anxiety-%E2%80%94-national-centre-mental-health (Accessed June 10, 2024).

[9] Charara R Forouzanfar M Naghavi M Moradi-Lakeh M Afshin A Vos T. The burden of mental disorders in the eastern Mediterranean region, 1990-2013. PLoS One. (2017) 12:e0169575. doi: 10.1371/journal.pone.0169575, PMID: 28095477 PMC5240956

[10] Baker DW. Health literacy and mortality among elderly persons. Arch Intern Med. (2007) 167:1503. doi: 10.1001/archinte.167.14.1503 17646604

[11] Tambling RR D’Aniello C Russell BS. Mental health literacy: a critical target for narrowing racial disparities in behavioral health. Int J Ment Health Addict. (2023) 21:1867–81. doi: 10.1007/s11469-021-00694-w, PMID: 34785992 PMC8582339

[12] Bonabi H Müller M Ajdacic-Gross V Eisele J Rodgers S Seifritz E. Mental health literacy, attitudes to help seeking, and perceived need as predictors of mental health service use. J Nerv Ment Dis. (2016) 204:321–4. doi: 10.1097/NMD.0000000000000488, PMID: 27015396

[13] Hurley D Swann C Allen MS Ferguson HL Vella SA. A systematic review of parent and caregiver mental health literacy. Community Ment Health J. (2020) 56:2–21. doi: 10.1007/s10597-019-00454-0, PMID: 31541315

[14] Taleb R Kassab N Kebbe A Kreidieh N. Mental health literacy of the Lebanese population (MHeLLP): a cross-sectional study. J Public Ment Health. (2020) 20:132–44. doi: 10.1108/JPMH-04-2020-0031/FULL/XML

[15] Oztas B Aydoğan A. Determination of mental health literacy levels of health professionals. J Psychiatr Nurs. (2021) 12:198–204. doi: 10.14744/phd.2021.43265

[16] Bennett H Allitt B Hanna F. A perspective on mental health literacy and mental health issues among Australian youth: cultural, social, and environmental evidence! Front Public Health. (2023) 11:1065784. doi: 10.3389/fpubh.2023.1065784, PMID: 36741953 PMC9891461

[17] Cotton SM Wright A Harris MG Jorm AF Mcgorry PD. Influence of gender on mental health literacy in young Australians. Aust. N. Z. J. Psychiatry. (2006) 40:790–6. doi: 10.1080/j.1440-1614.2006.01885.x, PMID: 16911755

[18] Furnham A Winceslaus J. Psychiatric literacy and the personality disorders. Psychopathology. (2012) 45:29–41. doi: 10.1159/000325885 22123514

[19] Hadjimina E Furnham A. Influence of age and gender on mental health literacy of anxiety disorders. Psychiatry Res. (2017) 251:8–13. doi: 10.1016/j.psychres.2017.01.089, PMID: 28189082

[20] Ratnayake P Hyde C. Mental health literacy, help-seeking behaviour and wellbeing in young people: implications for practice. Educ Dev Psychol. (2019) 36:16–21. doi: 10.1017/edp.2019.1

[21] Anbesaw T Asmamaw A Adamu K Tsegaw M. Mental health literacy and its associated factors among traditional healers toward mental illness in northeast, Ethiopia: a mixed approach study. PLoS One. (2024) 19:e0298406. doi: 10.1371/journal.pone.0298406, PMID: 38394100 PMC10889902

[22] Dmour HH Marashdeh MF Al-Zubi AK Obaisat MA Al-Alwan MM. Stigma of mental illness in Jordan. J. R. Med. Serv. (2020) 27:70–5. doi: 10.12816/0055469

[23] O’Connor M Casey L. The mental health literacy scale (MHLS): a new scale-based measure of mental health literacy. Psychiatry Res. (2015) 229:511–6. doi: 10.1016/j.psychres.2015.05.064, PMID: 26228163

[24] Alshehri E Alosaimi D Rufaidi E Alsomali N Tumala R. Mental health literacy scale Arabic version: a validation study among Saudi university students. Front Psych. (2021) 12:41146. doi: 10.3389/FPSYT.2021.741146, PMID: 34646177 PMC8502930

[25] BinDhim NF Althumiri NA Ad-Dab’bagh Y Alqahtani MMJ Alshayea AK Al-Luhaidan SM. Validation and psychometric testing of the Arabic version of the mental health literacy scale among the Saudi Arabian general population. Int J Ment Health Syst. (2023) 17:1–8. doi: 10.1186/S13033-023-00615-5/TABLES/2 38053169 PMC10696716

[26] Brislin RW. Back-translation for cross-cultural research. J Cross-Cult Psychol. (1970) 1:185–216. doi: 10.1177/135910457000100301

[27] Costello AB Osborne J. Best practices in exploratory factor analysis: four recommendations for getting the most from your analysis. Res. Eval. Pract. Assess. Res. Eval. (2005) 10:7. doi: 10.7275/jyj1-4868

[28] Fisher WP. Rating scale instrument quality criteria. Rasch Meas Trans. (2007) 21:1095.

[29] Karabatsos G. The Rasch model, additive conjoint measurement, and new models of probabilistic measurement theory. J Appl Meas. (2001) 2:389–423. PMID: 12011506

[30] Wright B Linacre J. Reasonable Mean-Square fit values. Rasch Meas Trans. (1994) 8:370.

[31] Zwick R Thayer DT Lewis C. An empirical Bayes approach to mantel-Haenszel DIF analysis. J Educ Meas. (1999) 36:1–28. doi: 10.1111/j.1745-3984.1999.tb00543.x

[32] Krohne N Gomboc V Lavrič M Podlogar T Poštuvan V Šedivy NZ. Slovenian validation of the mental health literacy scale (S-MHLS) on the general population: a four-factor model. Inquiry. (2022) 59:110471. doi: 10.1177/00469580211047193, PMID: 35135367 PMC8832589

[33] Heizomi H Kouzekanani K Jafarabadi MA Allahverdipour H. Psychometric properties of the Persian version of mental health literacy scale. Int J Womens Health. (2020) 12:513–20. doi: 10.2147/IJWH.S252348, PMID: 32753978 PMC7351619

[34] Wang A Jia S Shi Z Sun X Zhu Y Shen M. Validation and psychometric testing of the Chinese version of the mental health literacy scale among nurses. Front Psychol. (2022) 12:1883. doi: 10.3389/fpsyg.2021.791883, PMID: 35153915 PMC8826253

[35] Parsons CE Crane C Parsons LJ Fjorback LO Kuyken W. Home practice in mindfulness-based cognitive therapy and mindfulness-based stress reduction: a systematic review and meta-analysis of participants’ mindfulness practice and its association with outcomes. Behav Res Ther. (2017) 95:29–41. doi: 10.1016/J.BRAT.2017.05.004, PMID: 28527330 PMC5501725

[36] Corrigan PW Watson AC. The paradox of self-stigma and mental illness. Clin Psychol Sci Pract. (2002) 9:35–53. doi: 10.1093/CLIPSY.9.1.35

[37] Dwairy M Achoui M Abouserie R Farah A Sakhleh AA Fayad M. Parenting styles in Arab societies. J Cross Cult Psychol. (2006) 37:230–47. doi: 10.1177/0022022106286922

[38] Gearing RE MacKenzie MJ Ibrahim RW Brewer KB Batayneh JS Schwalbe CSJ. Stigma and mental health treatment of adolescents with depression in Jordan. Community Ment Health J. (2015) 51:111–7. doi: 10.1007/s10597-014-9756-1, PMID: 25027014

[39] Kutcher S Bagnell A Wei Y. Mental health literacy in secondary schools: a Canadian approach. Child Adolesc Psychiatr Clin N Am. (2015) 24:233–44. doi: 10.1016/J.CHC.2014.11.007 25773321

[40] Al-Krenawi A. Mental health practice in Arab countries. Curr Opin Psychiatry. (2005) 18:560–4. doi: 10.1097/01.YCO.0000179498.46182.8B 16639119

[41] Patel V Saxena S Lund C Thornicroft G Baingana F Bolton P. The lancet commission on global mental health and sustainable development. Lancet. (2018) 392:1553–98. doi: 10.1016/S0140-6736(18)31612-X, PMID: 30314863

[42] al-Sagarat AY al Hadid LA Tapsell A Moxham L al Barmawi M Khalifeh AH. Evaluating and identifying predictors of emotional well-being in nursing students in Jordan: a cross-sectional study. Adv Ment Health. (2022) 20:242–52. doi: 10.1080/18387357.2021.2018940

[43] Rana H Racha R Reham R. (n.d.). *Economic Research Forum (ERF)*. Available at: https://theforum.erf.org.eg/2021/07/13/inequality-income-education-jordan/ (Accessed February 26, 2024).

[44] Elyamani R Naja S Al-Dahshan A Hamoud H Bougmiza MI Alkubaisi N. Mental health literacy in Arab states of the Gulf cooperation council: a systematic review. PLoS One. (2021) 16:e0245156. doi: 10.1371/journal.pone.0245156, PMID: 33411793 PMC7790272

[45] Clement S Schauman O Graham T Maggioni F Evans-Lacko S Bezborodovs N. What is the impact of mental health-related stigma on help-seeking? A systematic review of quantitative and qualitative studies. Psychol Med. (2015) 45:11–27. doi: 10.1017/S0033291714000129, PMID: 24569086

[46] Mansfield R Patalay P Humphrey N. A systematic literature review of existing conceptualisation and measurement of mental health literacy in adolescent research: current challenges and inconsistencies. BMC Public Health. (2020) 20:607. doi: 10.1186/s12889-020-08734-1, PMID: 32357881 PMC7195735

[47] Schneider J. Community work – a cure for stigma and social exclusion? Psychiatr Bull. (2009) 33:281–4. doi: 10.1192/pb.bp.108.022343

[48] Clement S Lassman F Barley E Evans-Lacko S Williams P Yamaguchi S. Mass media interventions for reducing mental health-related stigma. Cochrane Database Syst Rev. (2013) 2013:CD009453. doi: 10.1002/14651858.CD009453.pub2, PMID: 23881731 PMC9773732

[49] Patel V Belkin GS Chockalingam A Cooper J Saxena S Unützer J. Grand challenges: integrating mental health services into priority health care platforms. PLoS Med. (2013) 10:e1001448. doi: 10.1371/JOURNAL.PMED.1001448, PMID: 23737736 PMC3666874

[50] Bawadi H Al-Hamdan Z Khader Y Aldalaykeh M. Barriers to the use of mental health services by Syrian refugees in Jordan: a qualitative study. East Mediterr Health J. (2022) 28:197–203. doi: 10.26719/emhj.22.030, PMID: 35394051

[51] Al-Soleiti M Abu Adi M Nashwan A Rafla-Yuan E. Barriers and opportunities for refugee mental health services: clinician recommendations from Jordan. Glob Ment Health. (2021) 8:e38. doi: 10.1017/gmh.2021.36, PMID: 34631114 PMC8482442

[52] Kutcher S Wei Y Coniglio C. Mental health literacy. Can J Psychiatry. (2016) 61:154–8. doi: 10.1177/0706743715616609, PMID: 27254090 PMC4813415

